# Clinician Education and Workflow Tools to Improve Access to Recurrent and Metastatic Cervical Cancer Treatment

**DOI:** 10.7759/cureus.103426

**Published:** 2026-02-11

**Authors:** Nancy L Belcher

**Affiliations:** 1 Public Health, King County Medical Society, Seattle, USA

**Keywords:** cancer, cervical cancer screening, education, health professional's education, ob-gyn, obstetrics and gynecology (ob-gyn), obstetrics & gynecology, provider education and collaboration, rare gynecologic cancer

## Abstract

Background: Patients with recurrent or metastatic cervical cancer often face delays in treatment due to complex prior authorization (PA) processes and uneven access to specialty care. Educational interventions targeting clinicians and care teams may help streamline workflows and improve access to timely, evidence-based treatment.

Objective: To describe the design, implementation, and outcomes of a multidisciplinary quality-improvement initiative aimed at improving provider knowledge, administrative efficiency, and access to cervical cancer care.

Methods: The King County Medical Society (KCMS) launched the Cervical Cancer Education and Equity Initiative. The project combined clinician education with practical workflow tools, including a freely available Prior Authorization Toolkit. Activities included 21 short educational videos, a comprehensive slide-based educational module, monthly newsletters, social-media dissemination, and a centralized web hub integrating resources on immunotherapy, targeted therapy, and biomarker-driven care. Eighty-three clinicians completed the baseline survey (N=83), and 30 clinicians completed the follow-up survey, 30/83 (36.1%). Post-intervention surveys assessed clinician knowledge, confidence, and perceived impact on practice.

Results: Across knowledge/confidence items, 26/30 (86.7%) respondents completed each rating question; mean post-program scores ranged from 3.7 to 4.0 out of 5.0. For post-intervention practice change items, respondents reported earlier referral to specialists, 11/25 (44.0%); more frequent discussion of clinical trial options, 6/25 (24.0%); and increased attention to transportation, financial, or social-support barriers when planning care, 10/25 (40.0%). Respondents reported considering social determinants of health more often after the program, 17/25 (68.0%); 4/25 (16.0%) reported no change, and 4/25 (16.0%) indicated the item was not applicable to their role. A total of 1,869 views were recorded across 21 on-demand educational videos during the program period. All educational materials remained publicly accessible.

Conclusions: Targeted, workflow-aligned education paired with practical administrative tools may reduce barriers to cervical cancer treatment. This initiative demonstrates a replicable model for improving access to advanced oncology care through clinician education and systems-focused interventions.

## Introduction

Cervical cancer remains a major public health challenge worldwide, with substantial morbidity and mortality despite advances in prevention and treatment. Global surveillance data continue to demonstrate a high burden of disease and persistent disparities across regions and populations [1-3]. Patients with recurrent/metastatic cervical cancer represent the highest-acuity segment of the disease continuum and experience the poorest outcomes, underscoring the need for timely access to evidence-based systemic therapy and specialty care [4].

Systemic treatment options for recurrent/metastatic cervical cancer have expanded in recent years. Randomized clinical trials have established immunotherapy-based regimens as a standard component of first-line management for eligible patients, and additional agents have increased options for later lines of therapy [5-7]. As the therapeutic landscape evolves, guideline-concordant care increasingly depends on timely referral to appropriate specialists, completion of recommended diagnostic workup (including biomarker testing when indicated), and alignment of treatment selection with the most current evidence and guidelines [4-7].

However, delays in treatment initiation remain common, and administrative barriers are a well-documented contributor. Prior authorization processes can postpone therapy, increase clinician and staff workload, and disrupt care delivery in oncology settings [8-12]. Such delays are clinically meaningful, given evidence that longer time to treatment initiation is associated with worse outcomes across cancer settings [13]. Administrative burden may also exacerbate inequities when patients experience medical financial hardship or other social determinants of health (SDOH) that interfere with accessing specialty services, completing required testing, and navigating complex care pathways [14,15].

Clinicians and care teams therefore benefit from concise, practical resources that integrate current clinical guidance with operational tools to support efficient authorization, referral, and care coordination workflows [16]. The Cervical Cancer Education and Equity Initiative was developed to address these gaps by pairing evidence-based clinician education with actionable workflow resources, including a Prior Authorization Toolkit, to support clinician-reported confidence and perceived navigation of administrative processes related to timely and equitable care for patients with recurrent/metastatic cervical cancer.

## Materials and methods

Study design and setting

The King County Medical Society (KCMS) conducted a regional educational quality-improvement initiative designed to support clinician-reported confidence and perceived navigation of evidence-based care for patients with recurrent/metastatic cervical cancer. The initiative ran from December 2024 through January 2026 and integrated clinician education, system-navigation resources, and broad digital dissemination to address clinical and administrative barriers to care, including prior authorization (PA) processes.

Intervention components

Educational content included a comprehensive slide-based module covering cervical cancer epidemiology, current treatment guidelines, biomarker and molecular testing, immunotherapy and targeted therapies, treatment sequencing, and PA workflows. This module served as the foundational source document for derivative educational materials, including downloadable clinical flyers and quick-reference tools. In addition, 21 short-form educational videos were produced and made publicly available via the KCMS website and the KCMS YouTube channel. Subject-matter experts reviewed all educational content for clinical accuracy and relevance.

Dissemination strategies included monthly electronic newsletters reaching more than 6,000 healthcare professionals, regular social media posts across multiple platforms, and a dedicated cervical cancer education page on the KCMS website housing all videos, downloadable materials, and toolkit components. All resources were freely accessible, with no registration required.

A downloadable Prior Authorization Toolkit was developed to support clinical teams in navigating oncology-related administrative requirements. The toolkit included step-by-step workflow guidance, documentation checklists, appeal templates, staff training resources, and printable clinic- and patient-facing materials. The toolkit was designed to promote standardized documentation practices and support perceived efficiency in administrative processes related to treatment initiation

Survey design and data collection

Program evaluation was conducted using baseline and follow-up surveys administered through SurveyMonkey. Survey items were developed by the authors for this program evaluation and had not been previously published, and were not formally validated. The instrument included items assessing respondent role and practice setting; awareness and use of KCMS educational resources; self-rated knowledge and confidence (5-point Likert-scale items); perceived program value; and self-reported perceived practice changes (select-all-that-apply and open-ended items). The complete survey instruments are provided in Appendix A and Appendix B; no external permissions were required.

The baseline survey was distributed via the KCMS newsletter and posted on the KCMS website. A total of 83 respondents completed the baseline survey (N=83). The follow-up survey was administered to baseline respondents and was open from December 1, 2025, through December 31, 2025. Of the 83 baseline respondents, 30 completed the follow-up survey (30/83 (36.1%); N=30). To reduce duplicate responses, SurveyMonkey settings restricted responses to one per device/browser.

Inclusion and exclusion criteria

Inclusion criteria were (1) self-identified healthcare professionals and/or members of clinical care teams and (2) voluntary completion of the baseline survey distributed through KCMS channels during the study period. For follow-up analyses, respondents were included if they completed both the baseline and follow-up surveys (30/83 (36.1%)).

Exclusion criteria were duplicate submissions when identifiable. Incomplete responses were excluded from analyses for the corresponding survey item; therefore, denominators vary by item and are reported for each outcome as n/N (%).

Outcomes

Primary outcomes included self-reported knowledge and confidence related to recurrent/metastatic cervical cancer care, including familiarity with biomarker-driven care; confidence in determining when referral to specialty or higher-level care may be indicated; confidence in navigating PA requirements; awareness of clinical trial options; and confidence supporting underserved populations and patients with limited English proficiency. Secondary outcomes included self-reported practice changes; perceived support in managing complex cases; emotional exhaustion related to managing recurrent/metastatic cervical cancer care; and helpfulness and satisfaction with specific KCMS resources (website materials, newsletters, videos, and the PA toolkit). Outcomes are reported as frequencies and percentages in the format n/N (%), with item-level denominators provided.

Data analysis

Survey responses were analyzed descriptively, consistent with the exploratory, perception-based aims of this quality-improvement initiative. Categorical responses are reported as frequencies and percentages (n/N (%)). Likert-scale ratings are summarized using means with the corresponding item denominator (n) reported. Open-ended responses were summarized descriptively.

Ethics and consent

This project involved human participants (clinicians) through a voluntary, anonymous program-evaluation survey administered via SurveyMonkey and distributed through KCMS channels. No patient data, live human tissue, or identifiable private information were collected or reported. Completion of the survey constituted implied informed consent. KCMS determined that this activity constituted an anonymous clinician-focused program evaluation/quality improvement project and that formal institutional review board (IRB) review was not required; therefore, IRB approval was not applicable.

## Results

Eighty-three clinicians completed the baseline survey (N=83). Respondents were primarily MD/DO 37/83 (44.58%), followed by physician assistant (PA) 17/83 (20.48%) and nurse practitioner (NP) 14/83 (16.87%); smaller proportions included registered nurse (RN) 3/83 (3.61%) and clinical nurse specialist (CNS) 2/83 (2.41%). An additional 10/83 (12.05%) selected “Other,” which included medical assistants and other office staff.

Clinicians represented a broad range of specialties, including internal medicine 18/83 (21.69%), family medicine 12/83 (14.46%), gynecologic oncology 9/83 (10.84%), obstetrics/gynecology (OB/GYN) 7/83 (8.43%), and medical oncology 2/83 (2.41%). “Other” specialty was selected by 35/83 (42.17%) and included write-in specialties such as radiology, orthopedics, emergency medicine, general surgery, anesthesia, and pathology.

Across baseline items assessing barriers to evidence-based care, respondents prioritized administrative/payer constraints, workload limitations, and equity-linked access challenges. For systemic barriers, prior authorization/insurance delays and denials were rated as most important (mean importance score 4.50; higher scores indicate greater importance), followed by insurance denial of treatment options (4.00) and patient financial constraints (3.50). Professional barriers were led by burnout/high work demands (4.25), with lack of time for professional development and geographic barriers rated similarly (3.67 each). Patient-related barriers were dominated by health disparities in underserved populations (5.25) and cultural/language barriers (4.67), followed by patient non-adherence (3.75) and limited culturally relevant education resources (3.67). Baseline confidence in providing effective care was moderate for patients with limited English proficiency (LEP) (mean 3.25/5; n=83) and underserved/marginalized patients (mean 3.00/5; n=83). Respondents also identified barriers to accessing advanced treatment options for underserved patients, including clinical trials.

Preferred educational formats favored practical, guideline-based resources; clinical guidelines were selected most frequently. These findings informed the development of the intervention materials, including a slide-based clinician education module and short-form videos addressing guideline-based management, biomarker testing, treatment sequencing, and clinical trial awareness, as well as a Prior Authorization Toolkit with workflow guidance, documentation checklists, and appeal templates designed to reduce administrative delays.

Post-intervention survey responses are summarized in Table 1. Respondents reported increased knowledge of current systemic treatment options for recurrent/metastatic cervical cancer, 20/26 (76.9%) (mean 4.0/5; n=26), and increased understanding of biomarker testing in treatment selection, 16/26 (61.5%) (mean 3.9/5; n=26). Increased confidence in determining when a patient should be referred to a specialist or higher-level center was reported by 16/26 (61.5%) (mean 3.9/5; n=26), and increased confidence in navigating prior authorization requirements was reported by 12/26 (46.2%) (mean 3.7/5; n=26). Awareness of clinical trial options increased for 14/25 (56.0%), was unchanged for 10/25 (40.0%), and decreased for 1/25 (4.0%).

**Table 1:** Educational outcomes and practice impact of the KCMS Cervical Cancer Education and Equity Initiative Data represent post-intervention, self-reported clinician survey responses collected anonymously via SurveyMonkey. Values are reported as n/N (%) unless otherwise noted; denominators vary by item due to incomplete responses. Mean change ratings are reported as the mean rating on a 5-point scale (1 = much less than before, 2 = a little less than before, 3 = no change, 4 = a little more than before, 5 = much more than before), with the corresponding sample size (n). For categorical change items, Increased combines response options 4–5 and Decreased reflects response option 2 (no respondents selected response option 1 for these items). Multi-select practice-change items allowed respondents to select more than one option; therefore, percentages may total >100%. “Not applicable” was included as a response option only for select items; where it was not offered, values are shown as 0/N (0.0%). Mean change ratings apply only to items assessed on the 5-point change scale; for items not assessed on that scale, the mean change rating column is populated as 0.0. KCMS = King County Medical Society; SDOH = social determinants of health

Outcome domain	Measure	N	Mean change rating (1–5)	Increased n/N (%)	No change n/N (%)	Decreased n/N (%)	More often n/N (%)	Selected n/N (%)	Not applicable n/N (%)
Clinician knowledge	Knowledge of current systemic treatment options	26	4.0	20/26 (76.9%)	6/26 (23.1%)	0/26 (0.0%)	0/26 (0.0%)	0/26 (0.0%)	0/26 (0.0%)
Clinician knowledge	Understanding of biomarker testing role	26	3.9	16/26 (61.5%)	10/26 (38.5%)	0/26 (0.0%)	0/26 (0.0%)	0/26 (0.0%)	0/26 (0.0%)
Clinical confidence	Confidence in referring to a specialist/higher-level center	26	3.9	16/26 (61.5%)	10/26 (38.5%)	0/26 (0.0%)	0/26 (0.0%)	0/26 (0.0%)	0/26 (0.0%)
Treatment navigation	Confidence in navigating prior authorization requirements	26	3.7	12/26 (46.2%)	14/26 (53.8%)	0/26 (0.0%)	0/26 (0.0%)	0/26 (0.0%)	0/26 (0.0%)
Referral awareness	Awareness of clinical trial options	25	3.8	14/25 (56.0%)	10/25 (40.0%)	1/25 (4.0%)	0/25 (0.0%)	0/25 (0.0%)	0/25 (0.0%)
Equity & SDOH	Consideration of SDOH when planning care	25	0.0	0/25 (0.0%)	4/25 (16.0%)	0/25 (0.0%)	17/25 (68.0%)	0/25 (0.0%)	4/25 (16.0%)
Care coordination	Practice change: Earlier specialist referral (multi-select)	25	0.0	0/25 (0.0%)	0/25 (0.0%)	0/25 (0.0%)	0/25 (0.0%)	11/25 (44.0%)	0/25 (0.0%)
Care coordination	Practice change: Greater attention to barriers (multi-select)	25	0.0	0/25 (0.0%)	0/25 (0.0%)	0/25 (0.0%)	0/25 (0.0%)	10/25 (40.0%)	0/25 (0.0%)

Respondents reported considering social determinants of health more often when planning care for recurrent/metastatic cervical cancer, 17/25 (68.0%). Reported practice changes included earlier referral to gynecologic or medical oncology specialists, 11/25 (44.0%), and increased attention to transportation, financial, or social-support barriers when planning care, 10/25 (40.0%).

Engagement metrics included 1,869 total views across selected educational videos during the program period. Resource formats were rated as moderately to very helpful (mean helpfulness ratings 3.2-3.7 out of 5; n=22), and educational materials remained publicly available on the KCMS website.

## Discussion

This regional quality-improvement initiative evaluated a clinician-focused education program paired with workflow tools intended to support clinician awareness, confidence, and perceived navigation of administrative and care-coordination processes related to recurrent or metastatic cervical cancer. Post-intervention survey responses indicated increased self-reported knowledge of systemic treatment options (mean change 4.0/5; increased 20/26 (76.9%)) and increased understanding of biomarker testing in treatment selection (mean change 3.9/5; increased 16/26 (61.5%)). Respondents also reported increased confidence in navigating prior authorization requirements (mean change 3.7/5; increased 12/26 (46.2%)) and increased confidence in determining when referral to a specialist or higher-level center was indicated (mean change 3.9/5; increased 16/26 (61.5%)). These findings reflect clinician-reported perceptions across domains relevant to guideline-concordant care in recurrent/metastatic cervical cancer, including biomarker-informed treatment selection, immunotherapy and targeted therapy use in appropriate patients, and timely referral to specialty care [4-7].

A pragmatic feature of the initiative was the availability of asynchronous educational content and downloadable tools through a centralized website hub. Participants rated KCMS resources as moderately to very helpful, with mean helpfulness ratings ranging from 3.2 to 3.7 out of 5 across formats (n=22). Video engagement during the program period totaled 1,869 views for the videos included in this analysis, indicating utilization of the educational assets, not necessarily educational effectiveness. The program’s emphasis on workflow tools is consistent with published literature describing prior authorization as a major source of administrative burden in oncology practice, including increased staff time, delays in care delivery, and disruption of clinical workflows [8-12].

Equity-related outcomes were also reported. Respondents indicated considering social determinants of health more often when planning care for recurrent/metastatic cervical cancer (17/25 (68.0%)); 4/25 (16.0%) reported no change, and 4/25 (16.0%) indicated the item was not applicable to their role. In addition, 10/25 (40.0%) reported paying more attention to transportation, financial, or social-support barriers when making treatment plans (multi-select item). These perception-based findings align with existing evidence that financial hardship and broader social determinants are associated with disparities in access to cancer care and outcomes [14,15]. Delays in treatment initiation are clinically meaningful, and observational evidence has demonstrated an association between longer time to initial cancer treatment and worse outcomes across cancer populations, underscoring the importance of reducing administrative barriers and improving care navigation [13]. Although delays in treatment initiation are known to be clinically meaningful, this initiative did not assess objective treatment timelines.

This initiative has limitations. Outcomes were based on voluntary, self-reported survey responses and did not include objective operational endpoints (e.g., prior authorization turnaround time, time to treatment initiation, or referral completion). Engagement metrics reflect resource utilization rather than individual-level learning outcomes. In addition, post-intervention responses were collected from a limited sample, and survey participation may reflect selection bias toward clinicians most interested in cervical cancer education or systems-navigation tools.

Despite these limitations, the initiative demonstrates the feasibility of disseminating clinician education alongside practical workflow resources in a real-world professional society setting. Continued public availability of materials may support ongoing utilization beyond the project period. Future evaluation could incorporate objective measures of access and timeliness (e.g., prior authorization turnaround time, referral completion, and time to treatment initiation) to assess whether clinician-reported perceptions correspond to measurable changes in care delivery [8-13].

## Conclusions

A clinician-centered educational program paired with practical workflow tools was followed by self-reported increases in clinician knowledge, confidence, and perceived care-navigation behaviors related to recurrent or metastatic cervical cancer. Post-intervention respondents reported perceived increases in knowledge of systemic treatment options, understanding of biomarker testing in treatment selection, confidence in determining when referral to specialty or higher-level care may be indicated, and confidence in navigating prior authorization requirements.

Freely accessible digital resources, including short educational videos, downloadable materials, and a Prior Authorization Toolkit, may represent a feasible approach for supporting clinician awareness of guideline-concordant oncology care and administrative processes. Professional medical societies may be well-positioned to disseminate timely clinical education and workflow tools through existing communication channels (e.g., newsletters and web hubs). Continued public availability of these resources may facilitate ongoing utilization beyond the intervention period and inform future adaptation, pending evaluation using objective outcome measures.

## Appendices

Appendix A

**Table 2:** Enhancing care for patients with recurrent/metastatic cervical cancer: baseline survey instrument (SurveyMonkey) Appendix A contains the full baseline survey instrument administered via SurveyMonkey for the KCMS Cervical Cancer Education and Equity Initiative. Items are numbered Q1–Q51 in the order presented to respondents. Question formats include single-select, multi-select, open-ended, Likert-scale, frequency-scale, and rank-order items. Some items include conditional follow-up fields (e.g., “Other (please specify)” and “If no, please explain”) that allow respondents to provide free-text responses to supplement fixed-choice options. The ZIP code item (Q8) is optional, and respondents may enter N/A for open-ended questions where indicated (e.g., Q39).

Item	Question	Response type	Response options/scale
Title	Enhancing Care for Patients with Recurrent/Metastatic Cervical Cancer: A Baseline Survey of Healthcare Practices and Needs	Text	Not applicable
Q1	What is your current role?	Select all that apply	Medical Doctor (MD) or Doctor of Osteopathic Medicine (DO); Physician Assistant/Associate (PA); Nurse Practitioner (NP); Clinical Nurse Specialist (CNS); Advanced Practice Nurse (APN); Nurse (RN); Pharmacist; Other (please specify: Health Educator, Program Coordinator, Medical Assistant, Nursing Aide, Therapist)
Q2	What is your best contact email address?	Open-ended	Text entry
Q3	What is your specialty?	Select one	Gynecologic Oncology; Medical Oncology; Obstetrics/Gynecology (OB/GYN); Family Medicine; Internal Medicine; Other (please specify)
Q4	What is your level of involvement with patients with metastatic cervical cancer?	Select one	I directly manage and treat these patients; I diagnose these patients but refer them to specialists with no further follow-up; I diagnose these patients, refer them to specialists, and provide ongoing care after specialist treatment; I see these patients after another professional has diagnosed them and refer them to specialists for treatment; I provide ongoing care for these patients after they have been treated by specialists; I do not see or treat these patients; I am not a healthcare professional
Q5	How long have you been in practice (out of training)?	Select one	0–5 years; 6–10 years; 11–15 years; 16–20 years; 21–30 years; More than 31 years; Other (please specify)
Q6	What type of practice setting are you in?	Select all that apply	Academic Medical Center; Community Hospital; Private Practice; Outpatient Clinic; Community Health Clinic; Research Institution; Government/Military Facility; Other (please specify)
Q7	Approximately how many patients with recurrent/metastatic cervical cancer do you personally manage or assist in treating in a typical year?	Select one	0–1 patients; 2–5 patients; 6–10 patients; 10+ patients
Q8	To help us identify regional concerns, please provide the ZIP code of your current facility. This question is optional, and your response will remain confidential.	Open-ended	Text entry
Q9	How confident are you in your familiarity with the most current treatment protocols and recent advances, including emerging treatment options, new clinical guidelines, investigational therapies, and recently published studies for managing recurrent/metastatic cervical cancer?	5-point Likert	Not at all confident; Slightly confident; Moderately confident; Very confident; Extremely confident
Q10	Which treatment options are you comfortable discussing with patients?	Select all that apply	Immunotherapy; Chemotherapy; Targeted therapy; Clinical trials; Palliative care; Other (please specify)
Q11	Do you feel confident in selecting the most appropriate treatment option for a patient with recurrent/metastatic cervical cancer based on their clinical profile?	Select one	Yes; No; Somewhat
Q12	What factors influence your decision to prescribe treatments for patients with recurrent/metastatic cervical cancer?	Select all that apply	Patient’s clinical profile; Cost/insurance coverage; Patient’s preferences; Side effect profile; Availability of clinical trials and novel therapies; Peer consultation; Institutional formulary restrictions; Other (please specify)
Q13	How confident are you in diagnosing and screening for recurrent/metastatic cervical cancer?	5-point Likert	Not at all confident; Slightly confident; Moderately confident; Very confident; Extremely confident
Q14	How confident are you in applying evidence-based guidelines to inform your treatment decisions for recurrent/metastatic cervical cancer?	5-point Likert	Not at all confident; Slightly confident; Moderately confident; Very confident; Extremely confident
Q15	How often do you participate in multidisciplinary tumor boards or case discussions regarding recurrent/metastatic cervical cancer?	5-point frequency	Never; Rarely; Sometimes; Usually; Always
Q16	Do you regularly collaborate with non-oncology specialties when managing patients with recurrent/metastatic cervical cancer?	Select one	Yes; No; Other (please specify)
Q17	Do you feel that you have adequate resources (support staff, educational materials, guidelines) to manage patients with recurrent/metastatic cervical cancer?	Select one	Yes; No
Q18	Thinking about all your patients with recurrent/metastatic cervical cancer, how confident are you that they are receiving optimal care based on current best practices?	5-point Likert	Not at all confident; Slightly confident; Moderately confident; Very confident; Extremely confident
Q19	How often do your patients experience adverse events during treatment for recurrent/metastatic cervical cancer?	5-point frequency	Never; Rarely; Sometimes; Usually; Always
Q20	In general, how confident are you in managing adverse events related to the treatment of recurrent/metastatic cervical cancer?	5-point Likert	Not at all confident; Slightly confident; Moderately confident; Very confident; Extremely confident
Q21	Please rank the following systemic barriers for all patients in order of significance based on your experience providing care for patients with recurrent/metastatic cervical cancer. 1 being the most significant and 5 being the least significant. If a barrier is not applicable to your experience, please select N/A.	Rank order	Delays and denials related to prior authorization and insurance coverage; Insurance denial of treatment options; Financial constraints faced by patients; Limited access to multidisciplinary care; Lack of clinical trial opportunities; N/A
Q22	Please rank the following professional barriers in order of significance based on your experience providing care for patients with recurrent/metastatic cervical cancer. 1 being the most significant and 5 being the least significant. If a barrier is not applicable to your experience, please select N/A.	Rank order	Lack of access to current treatment guidelines; Lack of CME/administrative support; Lack of time for professional development; Geographic barriers (e.g., distance to treatment centers, rural healthcare access); Burnout and high work demands; N/A
Q23	Please rank the following patient-related barriers for all patients in order of significance based on your experience providing care for patients with recurrent/metastatic cervical cancer. 1 being the most significant and 6 being the least significant. If a barrier is not applicable to your experience, please select N/A.	Rank order	Patient non-compliance with treatment or follow-up; Cultural or language barriers affecting communication and care; Health disparities in underserved populations; Lack of culturally relevant education resources; Wait times for appointments; Staffing and turnover; N/A
Q24	Do you serve underserved or marginalized populations? Underserved or marginalized populations are groups facing significant healthcare barriers due to socioeconomic, geographic, linguistic, or cultural factors.	Select one	Yes; No; Other (please explain)
Q25	If yes, what percentage of your patient population is considered underserved or marginalized?	Open-ended	Numeric entry or text entry
Q26	Do you encounter patients with Limited English Proficiency (LEP) in your practice?	Select one	Yes; No; Other (please specify)
Q27	If yes, how frequently do you encounter patients with LEP?	Select one	Rarely (less than once a month); Occasionally (1–5 times/mo); Frequently (6–15 times/mo); Very frequently (>15 times/mo); Other (please specify)
Q28	Which languages do your LEP patients primarily speak?	Select all that apply	Spanish; Chinese (Mandarin or Cantonese); Vietnamese; Russian; Tagalog; Somali; Amharic; Korean; Arabic; Other (please specify)
Q29	How confident are you in providing effective care to specifically LEP patients with recurrent/metastatic cervical cancer?	5-point Likert	Not at all confident; Slightly confident; Moderately confident; Very confident; Extremely confident
Q30	How confident are you in providing effective care to underserved or marginalized patient populations with recurrent/metastatic cervical cancer?	5-point Likert	Not at all confident; Slightly confident; Moderately confident; Very confident; Extremely confident
Q31	When thinking about underserved or marginalized patients, how confident are you in managing adverse events for those with recurrent/metastatic cervical cancer?	5-point Likert	Not at all confident; Slightly confident; Moderately confident; Very confident; Extremely confident
Q32	How confident are you in recognizing when underserved or marginalized patients need further specialist referral?	5-point Likert	Not at all confident; Slightly confident; Moderately confident; Very confident; Extremely confident
Q33	What challenges do you face in referring underserved or marginalized patients with recurrent/metastatic cervical cancer?	Select all that apply	Lack of insurance coverage or financial assistance for patients; Limited availability of specialists who accept uninsured or underinsured patients; Transportation barriers (e.g., long travel distances, lack of reliable transportation); Limited availability of language support services (e.g., interpreters, bilingual staff); Long wait times for specialist appointments; Cultural or language misunderstandings affecting patient comprehension and engagement; Patient mistrust of healthcare system or reluctance to seek further care; Difficulty coordinating care among multiple professionals; Lack of patient access to information about treatment options or clinical trials; Limited access to facilities offering advanced or specialized treatments; Other (please specify)
Q34	For your underserved or marginalized patients with recurrent/metastatic cervical cancer, how confident are you that they are receiving optimal care based on current best practices?	5-point Likert	Not at all confident; Slightly confident; Moderately confident; Very confident; Extremely confident
Q35	In your practice setting, how often do you observe that underserved or marginalized patients face additional delays or barriers in accessing clinical trials or advanced treatments compared to other populations?	5-point frequency	Never; Rarely; Sometimes; Usually; Always
Q36	Do you feel there are any gaps in your knowledge or training when it comes to managing patients with recurrent/metastatic cervical cancer?	Select one	Yes; No
Q37	Which educational resources and/or formats do you find most helpful for professional development?	Select all that apply	Online videos and web-based resources; Tumor boards; Case-based learning modules; Conferences; Written correspondence (flyers, brochures, infographics, posters, etc.); Webinars; Peer-reviewed journals; Books; Other (please specify)
Q38	How likely are you to participate in online educational modules or tumor boards on recurrent/metastatic cervical cancer?	5-point Likert	Will not engage; Unlikely; Neutral; Likely; Extremely likely
Q39	What challenges do you face in staying updated on emerging treatments for recurrent/metastatic cervical cancer? Answer N/A if not applicable.	Open-ended	Text entry
Q40	Do you feel that you have adequate resources (support staff, educational materials, guidelines) to manage patients through the referral process with recurrent/metastatic cervical cancer?	Select one plus conditional open-ended	Yes; No; If no, please explain (text entry)
Q41	How often do you feel burnout or emotional exhaustion when managing patients with recurrent/metastatic cervical cancer?	5-point frequency	Never; Rarely; Sometimes; Usually; Always
Q42	To what extent do you feel that burnout or emotional exhaustion affects your ability to provide empathetic care to patients with recurrent/metastatic cervical cancer, especially those with poor prognosis or high-acuity needs?	5-point frequency	Never; Rarely; Sometimes; Usually; Always
Q43	What tools, resources, or strategies would help alleviate burnout and improve professional well-being while managing patients with recurrent/metastatic cervical cancer?	Open-ended	Text entry
Q44	What are the most significant challenges you face in managing patients with recurrent/metastatic cervical cancer?	Open-ended	Text entry
Q45	Are there specific educational resources or advancements you would like to see regarding recurrent/metastatic cervical cancer?	Open-ended	Text entry
Q46	What types of data or information are most helpful for clinical decision-making in the care of these patients?	Open-ended	Text entry
Q47	How often do you encounter challenges with patient adherence to recommended follow-up care or lifestyle modifications, and what strategies do you find effective in improving adherence?	Open-ended	Text entry
Q48	What strategies do you find effective in addressing patient adherence to recommended follow-up care or lifestyle modifications?	Open-ended	Text entry
Q49	What tools or resources would help you improve care for patients with recurrent/metastatic cervical cancer in your practice?	Open-ended	Text entry
Q50	Are there any challenges or gaps not addressed in this survey that you would like to highlight?	Open-ended	Text entry
Q51	Do you have any additional suggestions for improving the management of recurrent/metastatic cervical cancer, both for patients and healthcare professionals?	Open-ended	Text entry

Appendix B 

**Table 3:** Enhancing care for recurrent or metastatic cervical cancer: post-education follow-up survey instrument (SurveyMonkey) Appendix B contains the full post-education follow-up survey instrument administered via SurveyMonkey for the KCMS Cervical Cancer Education and Equity Initiative. Items are numbered Q1–Q30 in the order presented to respondents. Question formats include single-select, multi-select, and open-ended items. Two standardized 5-point Likert scales are used: perceived change items (Q7–Q14), with response options 1 = Much less than before, 2 = A little less than before, 3 = No change, 4 = A little more than before, and 5 = Much more than before; and helpfulness ratings (Q21–Q25), with response options 1 = Not at all helpful, 2 = Slightly helpful, 3 = Moderately helpful, 4 = Very helpful, and 5 = Extremely helpful. Items that include “Other (please specify)” or “Please describe” allow respondents to provide free-text responses to supplement fixed-choice options. The ZIP code item (Q3) is optional.

Item	Question	Response type	Response options/scale
Title	Enhancing Care for Recurrent or Metastatic Cervical Cancer: Post-Education Follow-Up Survey	Text	Not applicable
Q1	What is your primary role in patient care?	Select one	Radiation oncologist; Surgical oncologist; Primary care physician; Advanced practice provider (NP/PA); Pharmacist; Nurse; Medical oncologist; Gynecologic oncologist; Other (please specify)
Q2	Practice setting	Select all that apply	Academic Medical Center; Community Hospital; Community Health Clinic / FQHC; Private Practice; Government or Military Facility; Other (please specify)
Q3	ZIP code of your primary practice location (optional)	Open-ended	Text entry
Q4	Which KCMS cervical cancer education resources have you used?	Select all that apply	Slide deck / downloadable educational materials; KCMS newsletter items related to metastatic cervical cancer; KCMS website page on recurrent/metastatic cervical cancer; Short educational videos on recurrent/metastatic cervical cancer; Prior Authorization Toolkit (checklists, templates, workflow tools); None of the above; I am not sure; Other (please specify)
Q5	Approximately how many of the KCMS short educational videos have you watched?	Select one	1–2; 3–5; 6–10; More than 10; I have not watched the videos
Q6	How often have you referred to the Prior Authorization Toolkit in the past 6 months?	Select one	Never; Once; A few times; Monthly; More than once per month; Not applicable to my role
Q7	My knowledge of current systemic treatment options for recurrent/metastatic cervical cancer (e.g., immunotherapy, targeted therapy).	5-point Likert perceived change	1 = Much less than before; 2 = A little less than before; 3 = No change; 4 = A little more than before; 5 = Much more than before
Q8	My understanding of biomarker testing and its role in treatment selection.	5-point Likert perceived change	1 = Much less than before; 2 = A little less than before; 3 = No change; 4 = A little more than before; 5 = Much more than before
Q9	My confidence in determining when a patient with recurrent/metastatic cervical cancer should be referred to a specialist or higher-level center.	5-point Likert perceived change	1 = Much less than before; 2 = A little less than before; 3 = No change; 4 = A little more than before; 5 = Much more than before
Q10	My confidence in navigating prior authorization requirements for advanced cervical cancer treatments.	5-point Likert perceived change	1 = Much less than before; 2 = A little less than before; 3 = No change; 4 = A little more than before; 5 = Much more than before
Q11	My awareness of clinical trial options for patients with recurrent/metastatic cervical cancer.	5-point Likert perceived change	1 = Much less than before; 2 = A little less than before; 3 = No change; 4 = A little more than before; 5 = Much more than before
Q12	My ability to recognize and manage common adverse events associated with immunotherapy or targeted therapies.	5-point Likert perceived change	1 = Much less than before; 2 = A little less than before; 3 = No change; 4 = A little more than before; 5 = Much more than before
Q13	My confidence in supporting underserved or marginalized patients with recurrent/metastatic cervical cancer (e.g., those facing financial, transportation, or social barriers).	5-point Likert perceived change	1 = Much less than before; 2 = A little less than before; 3 = No change; 4 = A little more than before; 5 = Much more than before
Q14	My confidence in communicating with and supporting patients with limited English proficiency (LEP) about their diagnosis, treatment options, and referrals.	5-point Likert perceived change	1 = Much less than before; 2 = A little less than before; 3 = No change; 4 = A little more than before; 5 = Much more than before
Q15	Please share one example (if any) of how KCMS educational materials changed your understanding or approach to care for patients with recurrent/metastatic cervical cancer.	Open-ended	Text entry
Q16	As a result of the KCMS cervical cancer education, have you made any of the following changes?	Select all that apply plus open-ended	I refer patients earlier to gynecologic or medical oncology specialists; I discuss immunotherapy or targeted therapy options more consistently with eligible patients; I order or confirm appropriate biomarker testing more consistently; I am more likely to consider or discuss clinical trial options; I use a more structured workflow for prior authorization (checklists, templates, standard letters); I involve a multidisciplinary team (e.g., tumor board, palliative care, navigation) more often; I pay more attention to transportation, financial, or social-support barriers when making treatment plans; I routinely use interpreters or translated materials with LEP patients; No specific changes yet, but I intend to change my practice; Please describe (open-ended)
Q17	Overall, how much has the KCMS cervical cancer education improved your ability to provide high-quality care to patients with recurrent/metastatic cervical cancer?	Select one	Not at all; A little; Moderately; Quite a bit; A great deal
Q18	Since participating in the KCMS program, how often do you consider social determinants of health (e.g., transportation, cost, housing, caregiving) when planning care for patients with recurrent/metastatic cervical cancer?	Select one	Less often than before; About the same as before; Somewhat more often; Much more often; Not applicable to my role
Q19	To what extent did KCMS resources (videos, toolkit, tumor boards, conference) help you feel more supported in managing complex cervical cancer cases?	Select one	Not at all; A little; Moderately; Quite a bit; A great deal
Q20	Compared with before, how would you rate your current level of emotional exhaustion related specifically to managing recurrent/metastatic cervical cancer care?	Select one	Much higher; A little higher; About the same; A little lower; Much lower
Q21	Newsletter updates	5-point Likert helpfulness	1 = Not at all helpful; 2 = Slightly helpful; 3 = Moderately helpful; 4 = Very helpful; 5 = Extremely helpful
Q22	Committed KCMS Website for all cervical cancer materials	5-point Likert helpfulness	1 = Not at all helpful; 2 = Slightly helpful; 3 = Moderately helpful; 4 = Very helpful; 5 = Extremely helpful
Q23	Website and downloadable materials	5-point Likert helpfulness	1 = Not at all helpful; 2 = Slightly helpful; 3 = Moderately helpful; 4 = Very helpful; 5 = Extremely helpful
Q24	Prior Authorization Toolkit	5-point Likert helpfulness	1 = Not at all helpful; 2 = Slightly helpful; 3 = Moderately helpful; 4 = Very helpful; 5 = Extremely helpful
Q25	Short educational videos on YouTube	5-point Likert helpfulness	1 = Not at all helpful; 2 = Slightly helpful; 3 = Moderately helpful; 4 = Very helpful; 5 = Extremely helpful
Q26	Overall, how satisfied are you with the KCMS Cervical Cancer Education and Equity Initiative?	Select one	Very dissatisfied; Dissatisfied; Somewhat dissatisfied; Neutral; Somewhat satisfied; Satisfied; Very satisfied
Q27	What challenges do you still face in staying updated on the latest treatment protocols for recurrent/metastatic cervical cancer?	Open-ended	Text entry
Q28	What other tools, strategies, or resources could improve your ability to care for these patients?	Open-ended	Text entry
Q29	What topics would you most like further education on?	Open-ended	Text entry
Q30	Lastly, is there anything you would suggest to help KCMS improve this initiative or future programs?	Open-ended	Text entry
